# Egypt's Premier Medical Student Research Group: A New Model for Medical Student Research in Developing Countries

**DOI:** 10.7759/cureus.3561

**Published:** 2018-11-08

**Authors:** Ahmed S Negida

**Affiliations:** 1 Neurosurgery, Zagazig University, Zagazig, EGY

**Keywords:** medical student research, medical education, evidence based medicine

## Abstract

The involvement of medical students in scientific research has been widely advocated over the last decades in order to fulfill the need for a new generation of physicians who can apply evidence-based medicine. Efforts to involve medical students in scientific research have been described by individual institutions. In developing countries, students are less involved in research owing to the limited time and resources. Hereby, we describe our three-year experience with a novel model for medical student research in Egypt.

## Editorial

The involvement of medical students in scientific research has been widely advocated over the last decades. Educational institutions are preparing generations of scientist physicians who can advance medical sciences and bridge the gap with clinical practice. Additionally, the evolution of evidence-based medicine has changed the face of medical practice and medical education. Paul Glasziou stated that "a twenty-first century's clinician who cannot critically read a research paper is as unprepared as someone who cannot examine the cardiovascular system and measure the blood pressure" [1].

In developed countries, many educational institutions have involved student research into their educational program [2-4]. However, in developing countries, few reports have described the involvement of medical students in research. Common barriers towards student research in Egypt are lack of time, resources, and training [5]. Hereby, we report our experience in the Medical Research Group of Egypt (MRGE, www.negida.com/teams/mrge-egypt/). MRGE was established in 2014 as a non-governmental, non-profit, online-based, nationwide network of medical students across all Egyptian universities.

MRGE provides medical students with a free online training of (1) database and literature search, (2) clinical study designs, (3) basics of biostatistics, (4) evidence synthesis, (5) critical appraisal, and (6) scientific writing and academic publishing. Additional training is provided through our regular interactive, hands-on workshops in different Egyptian universities.

**Figure 1:**
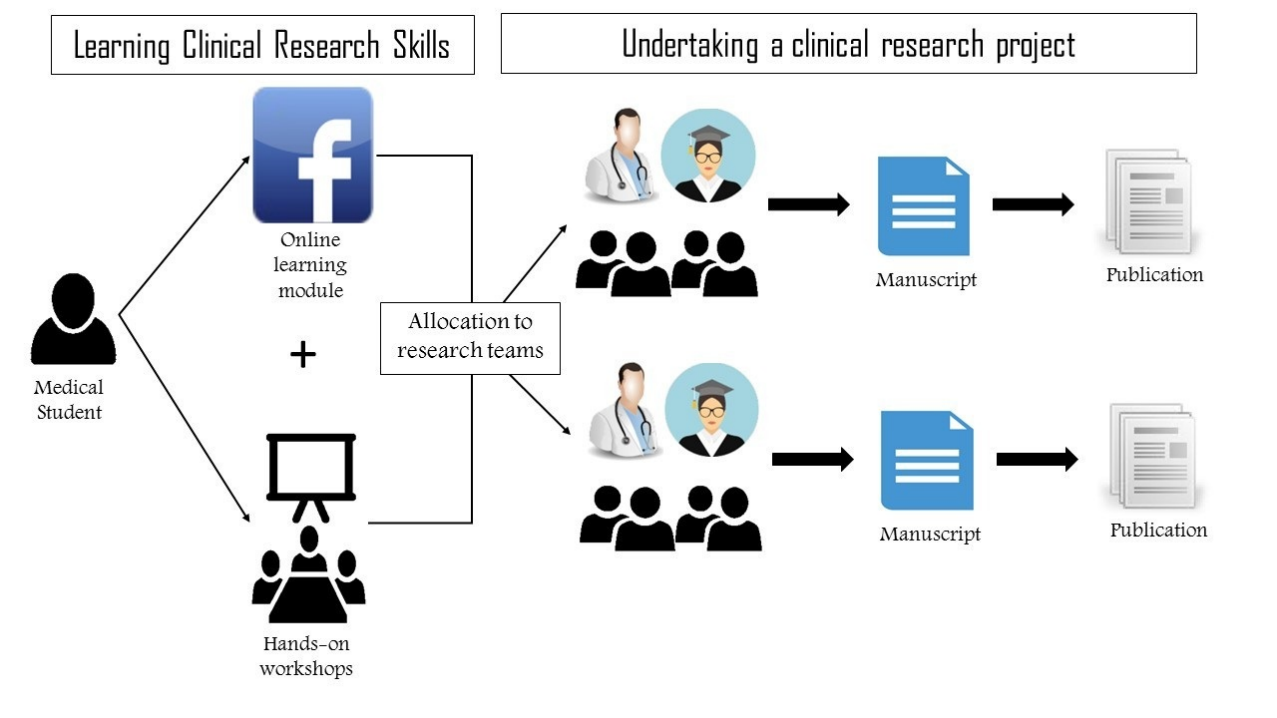
Flow diagram A flow diagram of the two phases of the medical student research model of the Medical Research Group of Egypt

Successful students who complete our training are matched into student interest groups working on an evidence synthesis project or a review of the literature. Research teams should include at least one senior student with experience in research methodology and a clinician or faculty member to provide the clinical expertise for the research team (Figure 1).

Since this model was initiated in 2014, 300 medical students from all the Egyptian universities have joined MRGE, 400 students were trained through our hands-on workshops, and other 1000 students joined our online learning module. This model has yielded several scientific publications, mostly systematic reviews, meta-analyses, and traditional review articles in many medical fields, including, but not limited to, neurology, oncology, hepatology, virology, and surgery. Interestingly, over time, hardworking students have the chance to take their collaboration up to participation in primary clinical research projects (for example, clinicaltrials.gov registration no: NCT02785510 and NCT02772744) and performing basic science experiments.

We recommend the replication of our non-governmental, online-based, student-led model in other developing countries.
